# Survey data on political attitudes of China׳s urban residents compiled from the Chinese General Social Survey (CGSS)

**DOI:** 10.1016/j.dib.2018.08.146

**Published:** 2018-08-30

**Authors:** Geng Niu, Guochang Zhao

**Affiliations:** Research Institute of Economics and Management, Southwestern University of Finance and Economics, China

## Abstract

The data discussed in this article is related to the article entitled “*Identity and trust in government: A comparison of locals and migrants in urban China*” (Niu and Zhao, 2018) [1], which analyses the political trust among three groups of urban residents in China (rural–urban migrants, urban–urban migrants, and urban locals) based on a sample with 4059 observations extracted from the 2010 Chinese General Social Survey (CGSS). The dataset contains detailed information on respondents’ political and social attitudes, demographic characteristics, and socio-economic status.

**Specifications Table**

**Table:** 

Subject area	*Economics, politics and sociology*
More specific subject area	*Political trust, hukou identity and socio-economic status*
Type of data	*Table and graph*
How data was acquired	*The authors acquired the data from the CGSS official website through registration.*
Data format	*Aggregated, analysed, and filtered*
Experimental factors	*The data was based on the CGSS dataset and was extracted using STATA and reorganized using the Stata **tabstat** and **ologit** package.*
Experimental features	*The data was collected from a survey that employed a stratified multi-stage probability proportional to size (PPS) sampling design.*
Data source location	*China*
Data accessibility	*The data was available from the Chinese National Survey Data Archive*http://cnsda.ruc.edu.cn
Related research article	G. Niu and G. Zhao, Identity and trust in government: A comparison of locals and migrants in urban China, Cities. (2018), https://doi.org/10.1016/j.cities.2018.06.008

**Value of the data**

• The dataset contains detailed information on social and political attitudes (in particular, political trust in the central and local governments), demographic characteristics, and socio-economic indictors.
• The dataset can be used to explore the distribution and determinants of political attitudes in urban China among groups with different social status.
• The dataset can be used by both researchers and policy makers to understand social attitudes in urban China.

## 1 Data

The data is based on the 2010 Chinese General Social Survey (CGSS), which is a national representative survey dataset. The original CGSS data consists of respondents from both rural and urban areas in China. The data used in this article only includes respondents residing in urban areas. The detailed sample selection procedure is provided below.

## 2 Experimental design, materials, and methods

### 2.1 Survey design

The CGSS is the first national continuous social survey project in mainland implemented by academic institutions, which can be considered as the Chinese counterpart of the General Social Survey (GSS) in the US. The project was jointly initiated in 2003 by Renmin University of China (RUC) and Hong Kong University of Science and Technology. The main objectives of the CGSS are to collect data on social trends in mainland China and to monitor the attitudes of Chinese people.^1^ As a non-official survey project, the CGSS asked for voluntary participation. Respondents were informed of the objectives and purposes of the survey projects before they began. In addition, the survey team signed written promises to keep personally identifiable information confidential. Above all, the CGSS adhered to high ethical and scholarly standards and has been used widely in social science research on China. See Bian and Li [2] for a more detailed introduction of the CGSS project.

A stratified multi-stage probability proportional to size (PPS) sampling design was employed by the 2010 CGSS. The sampling frame was the 2009 national population data. Samples were drawn from households in all 31 provincial units in mainland China. In a selected county (area), four community-level units (neighbourhood committees or village committees) were randomly selected. In a selected community-level unit, 25 households were sampled with the PPS method. In total 12,000 respondents in 400 community-level units were selected for the survey.

The 2010 CGSS collected information in various areas of Chinese people׳s demographic backgrounds, attitudes and behaviours, and socio-economic status, among others. Compared to the CGSS in other years, the 2010 CGSS captured a more comprehensive picture of respondents’ political attitudes.

### 2.2 Sample selection

The CGSS 2010 consisted of 11,783 valid respondents. We excluded 4561 respondents residing in rural areas since we focused on cities. Then, we divided the urban sample into three groups of people based on the household registration (*hukou*) status: rural migrants, urban migrants, and urban locals. Rural migrants had rural *hukou* from the countryside, urban migrants had urban *hukou* in cities other than the one where they resided at the time of the survey, and urban locals had local urban *hukou*. Furthermore, we restricted our sample to respondents aged between 16 and 60 years to facilitate comparison between migrants and locals. Finally, we dropped observations with missing values on dependent variables and explanatory variables. The final working sample consisted of 4059 respondents: 2601 urban locals, 1154 rural migrants, and 304 urban migrants.

### 2.3 Data measurements and variable definition

The data includes two variables on political trust. The first variable measures a respondent׳s trust in China׳s central government and the second variable measures trust in the local governments. Both variables are coded on a discrete scale from one (not at all) to five (very much). In addition, the data contains a variety of variables about respondents’ urban experience and social/political attitudes, including “unjustified treatment”, “perceived fairness”, “external efficacy”, “internal efficacy”, and “authority orientation”. The data also includes some variables that capture respondents’ demographic background and socio-economic attitudes: age, gender, education level, membership in the Communist Party of China (CPC), and household income per capita. Finally, the data includes city dummy variables that equal one for respondents residing in a given city. The explanations of those variables can be found in Niu and Zhao [1]. Table 1 presents descriptive statistics of the dataset. Table 2 summarizes levels of political trust in different groups of respondents.

**Table 1:** Descriptive statistics of the dataset.

	Mean	Std. Deviation	Max	Min
Trust in the central government	4.22	0.86	1	5
Trust in the local governments	3.59	1.07	1	5
Unfair treatment	0.09	0.29	0	1
Perceived fairness	2.82	1.07	1	5
Generalized trust	3.37	1.11	1	5
Internal efficacy	2.84	0.82	1	5
External efficacy	2.76	0.75	1	5
Authority orientation	2.72	0.51	1	5
Age	43.69	9.97	22	60
Female	0.53	0.50	0	1
Education level				
Junior high or below	0.42	0.49	0	1
Senior high	0.3	0.46	0	1
College or above	0.28	0.45	0	1
CPC member	0.14	0.35	0	1
Household income per capita (Yuan)	17,826.93	26,043.86	0	440,000

**Table 2:** Levels of political trust in different groups.

	Trust in the central government		Trust in the local governments	
	Mean	Std. Deviation	Mean	Std. Deviation
*Hukou* status				
Rural migrants	4.34	0.8	3.53	1.14
Urban migrants	3.94	0.97	3.38	1.04
Urban locals	4.2	0.85	3.64	1.03
Age group				
Age< 40	4.1	0.89	3.53	1.05
Age≥ 40	4.29	0.83	3.61	1.08
Gender				
Male	4.24	0.86	3.55	1.09
Female	4.21	0.85	3.62	1.05
Education				
Junior high or below	4.34	0.84	3.57	1.14
Senior high	4.21	0.82	3.61	1.01
College or above	4.05	0.89	3.58	1.01
CPC member				
No	4.22	0.87	3.57	1.08
Yes	4.26	0.78	3.71	0.96
Household income per capita				
< 10,000 Yuan	4.33	0.8	3.59	1.11
≥ 10,000 Yuan	4.14	0.88	3.58	1.04

### 2.4 Data description

See Table 1 and Table 2.

### 2.5 Method

With this data it was possible to employ a multivariate regression model to identify the determinants of political trust in urban China, as is the case in Niu and Zhao [1]. As political trust was measured on a discrete scale from one to five, an ordered logistic model could be used to analyse the determinants of political trust, as explained in Winship and Mare [3]. Empirical analysis could be performed using software such as Stata.

## Footnotes

1 http://www.chinagss.org/index.php?r=index/index&hl=en
