# Supplementary material for: Survey data on political attitudes of China׳s urban residents compiled from the Chinese General Social Survey (CGSS)
Source: Data Brief. 2018 Aug 30;20:591–5. doi: 10.1016/j.dib.2018.08.146 (PMC6127978; doi:10.1016/j.dib.2018.08.146)
Supplement: Supplementary file 2 — Supplementary material [file mmc2.docx]

AUTHOR DECLARATION

To whom it may concern,

We wish to confirm that there are no known conflicts of interests associated with this data in brief article.

Sincerely,

Geng Niu

Guochang Zhao
